# Transcriptomic analyses reveal physiological changes in sweet orange roots affected by citrus blight

**DOI:** 10.1186/s12864-019-6339-0

**Published:** 2019-12-11

**Authors:** Shimin Fu, Jonathan Shao, Avijit Roy, Ronald H. Brlansky, Changyong Zhou, John S. Hartung

**Affiliations:** 1 Citrus Research Institute, Southwest University, Chongqing, China; 2 United States Department of Agriculture-Agricultural Research Service, Molecular Plant Pathology Laboratory, Beltsville, MD USA; 3 USDA-APHIS-PPQ-S&T, Beltsville, MD 20705 USA; 4 Citrus Research and Education Center, University of Florida, Lake Alfred, FL 33850 USA

**Keywords:** Drought response, Water transport, Plant defense, Pathogenesis

## Abstract

**Background:**

Citrus blight is a very important progressive decline disease of commercial citrus. The etiology is unknown, although the disease can be transmitted by root grafts, suggesting a viral etiology. Diagnosis is made by demonstrating physical blockage of xylem cells that prevents the movement of water. This test was used to identify symptomatic trees from four commercial groves in Florida. Total RNA extracts of phloem-enriched scaffold root tissues were prepared from seven trees that failed to take up water and from one healthy tree. These RNA extracts were used for transcriptomic analyses using paired end RNA-Seq from an Illumina 2500 system. The expression of transcripts annotated as polyprotein of citrus endogenous pararetrovirus were estimated by both RT-qPCR and RNA-Seq.

**Results:**

Transcripts from seven RNA-Seq libraries from trees affected by citrus blight were compared to a control tree. 129–148 million RNA fragments (two paired-end reads/fragment) were generated per library and were mapped to the sweet orange reference genome. In response to citrus blight stress, genes encoding aquaporins, proteins with water channel activity and several cellulose synthase genes were down-regulated, whereas genes involved in lignin and glucosinolate biosynthesis were up-regulated. Transcripts encoding proteins in pathways of carbohydrate metabolism, nucleotide synthesis, signaling, hormone metabolism, secondary metabolism, transport, and biotic stress pathways were overwhelmingly down regulated in all libraries.

**Conclusion:**

Reduced water intake and xylem plugging were observed in the trees tested and the changes in their transcriptome were analyzed. Plants adapted to reduced water flow by regulating primary and secondary metabolism, nuclear transport and hormone associated pathways. The patterns of energy generation, transcription, translation and protein degradation were consistent with irreversible decline. The down regulation of cellulose synthase transcripts and up regulation of transcripts related to lignin production likely lead to an imbalance in the pathways leading to wood formation, and may lead to the blockage of the xylem vessels seen as the cardinal symptom of citrus blight. Transcripts of a pararetrovirus were elevated in the transcriptome of roots used in this study.

## Introduction

Citrus blight (CB) was described in Florida more than a century ago and 50 years of research on CB was reviewed in 1936 [41] and more recently [16]. CB is found in tropical or subtropical regions, notably Florida and Brazil but including the Caribbean, and portions of South Africa and Australia [9, 29]. CB is noticeably absent from California, Texas and Asia. The disease affects only bearing trees, primarily grapefruit (*Citrus paradisi* Macf.) and sweet orange (*Citrus sinensis* L.) and has not been reported in greenhouse grown trees. Symptoms begin with a distinct loss of reflective sheen on the surface of leaves, a mild wilt, and zinc-deficiency symptoms in the foliage. Trees rapidly decline with extensive twig dieback, small fruit and off-season flowering [3, 14] and levels of zinc become elevated in the wood [51]. CB has caused economic losses in excess of $60 million in Florida and was formerly the most significant plant health problem of Florida citrus with a loss of 650,000 trees/year [48], but with the unprecedented crisis of huanglongbing (HLB) throughout the world, research efforts on CB have diminished.

Trees affected with blight will not recover from the disease if they are severely pruned, which can temporarily reduce the symptoms of HLB. Trees affected by both HLB and CB may decline and die more rapidly than when affected by either disease alone, since the physiology of both the phloem (HLB) and xylem (CB) are disrupted simultaneously. Transmission of blight has been demonstrated by root grafts [37, 46, 49], but not by bud or approach grafts or through soil [47]. Efforts to use epidemiological models to gain insight into whether CB is a disease or a physiological disorder have been inconclusive, as the models provide conflicting results. In one study CB spread within a grove following a linear model not typical of a pathogen and vectored disease [12], but in another study a logistic model typical of a vector transmitted disease was observed at high levels of disease [11]. Several causal agents for CB have been proposed, including *Xylella fastidiosa*, *Fusarium solani*, and an idaeovirus [17, 23, 27] as well as some abiotic factors, but none of these causes have been confirmed and the etiology of the disease remains unknown. Therefore, neither the isolation of a pathogen nor the detection of its presence in pre-symptomatic trees with PCR-based methods are possible. Confirmatory diagnosis of the disease is primarily based on demonstration of the blockage of water uptake by attempting to inject water into the trunks of affected trees by using a drill to make a hole in the trunk and a syringe to inject the water [33]. Water cannot be injected into the trunk of trees affected by CB. The presence of amorphous plugs [8, 9] that physically fill the lumen of the xylem cells and measurement of elevated levels of zinc and soluble phenolics in the wood [51] are also used for diagnosis of CB. An immunoassay for a protein associated with citrus blight in bark tissues has also been used for presumptive diagnosis [6]. Recently, pararetrovirus transcripts have been found in both roots and leaves of blighted trees through Next Generation Sequencing (NGS) [43].

Because the etiology of CB remains unknown and there are no control methods or therapeutic treatments for CB, there is an urgent need for new data to provide both clues to a potential etiological agent and to provide insight that may lead to improved control and therapeutic measures. RNA-Seq has been widely used to provide insight into the pathophysiology of different plant diseases, including CB [55]. RNA-Seq has been particularly useful in the case of HLB and citrus sudden death disease, the causal agents of which are nonculturable [2, 19, 20, 38]. Distinct plant defense responses can be revealed, with different patterns of regulation in response to different pathogens and abiotic stress. We wanted to know how alterations in the transcriptome correspond with the symptoms and physiological changes in advanced stages of CB.

## Results

### Demonstration of xylem plugging in blight-affected trees

**Fig. 1:**
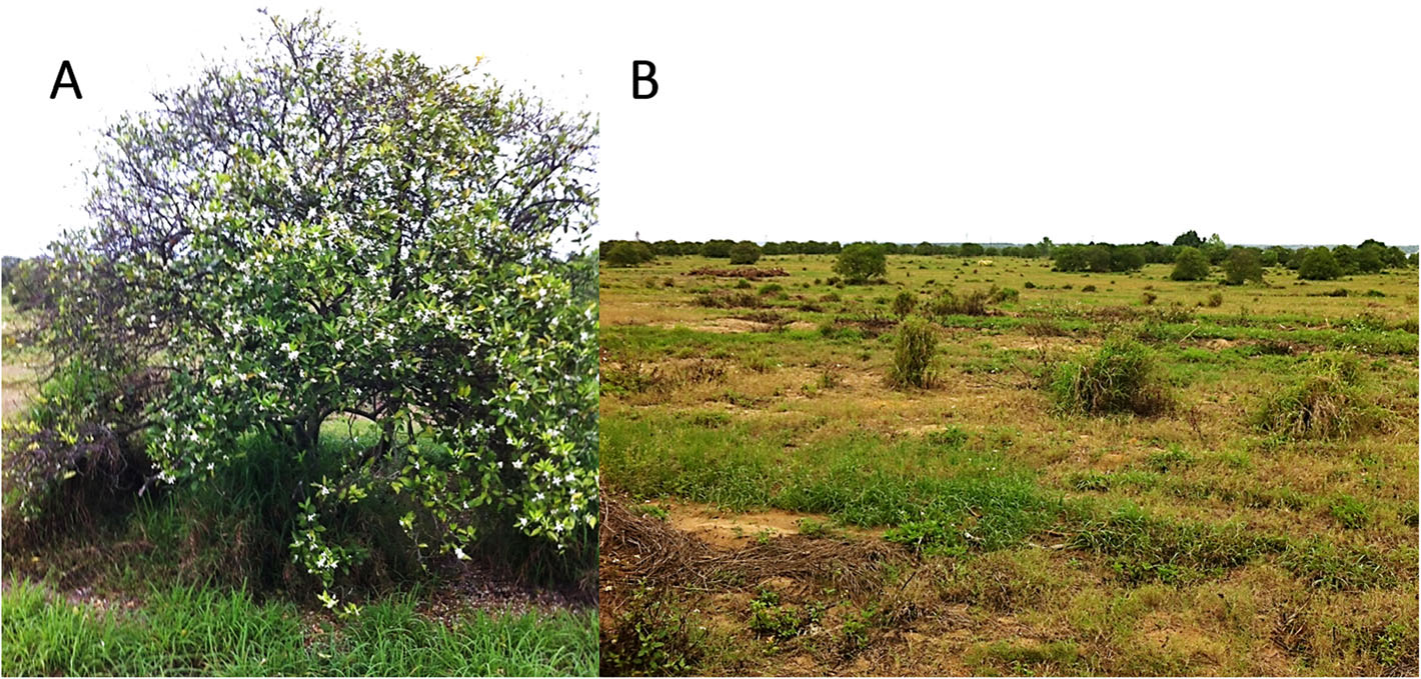
Dieback symptoms and complete loss of sweet orange grove affected by citrus blight. **a**, Severe dieback symptoms observed on tree diagnosed with citrus blight by the failure of water uptake assay; **b**, Near complete loss of sweet orange grove due to citrus blight. Only few and widely scattered trees remain in production

Symptoms observed on the CB affected trees included severe twig dieback and defoliation, as seen on Hamlin sweet orange grafted on citrumelo rootstock (*X Citroncirus spp*.) (Fig. 1a). In one of the locations, very few trees in the citrus block remained for sampling, as the other trees had already been removed due to the severity of the citrus blight problem (Fig. 1b). The ability of the trees to take up water into their trunks by syringe injection was measured to select trees from which root samples were taken to prepare RNA extracts. Five of the seven trees sampled failed to take up any water in the assay, and the remaining two trees took up only 4 and 10 ml compared to more than 15 ml observed in healthy trees in a 30 s test (Table 1) [33].

**Table 1:** Characteristics of eight RNA-Seq libraries used for transcriptome analysis

Library	# Fragments				Water uptake (ml/30s)	RIN
	Total	Mapped	Unmapped	Mapped/Total (%)		
HC	147,805,559	140,545,448	7,260,111	95.1	> 15	8.0
IM33R	129,158,036	107,984,108	21,173,928	83.6	0	3.4
DG49R	135,076,469	98,028,045	37,048,424	72.6	0	4.1
PC24R	134,734,532	127,706,788	7,027,744	94.8	10	3.5
PC26R	136,760,943	112,230,256	24,530,687	82.1	4	4.4
DG43S	133,565,526	56,889,420	76,676,106	42.6	0	1.0
DG50R	138,068,924	76,809,852	61,259,072	55.6	0	1.0
IM39R	131,665,044	63,225,754	68,439,290	48.0	0	2.2

*HC* healthy control; The rest of libraries were affected by citrus blight. Sequence reads were trimmed to remove low quality bases at the ends of reads

### Overview of the RNA-Seq data and transcriptome analysis

**Fig. 2:**
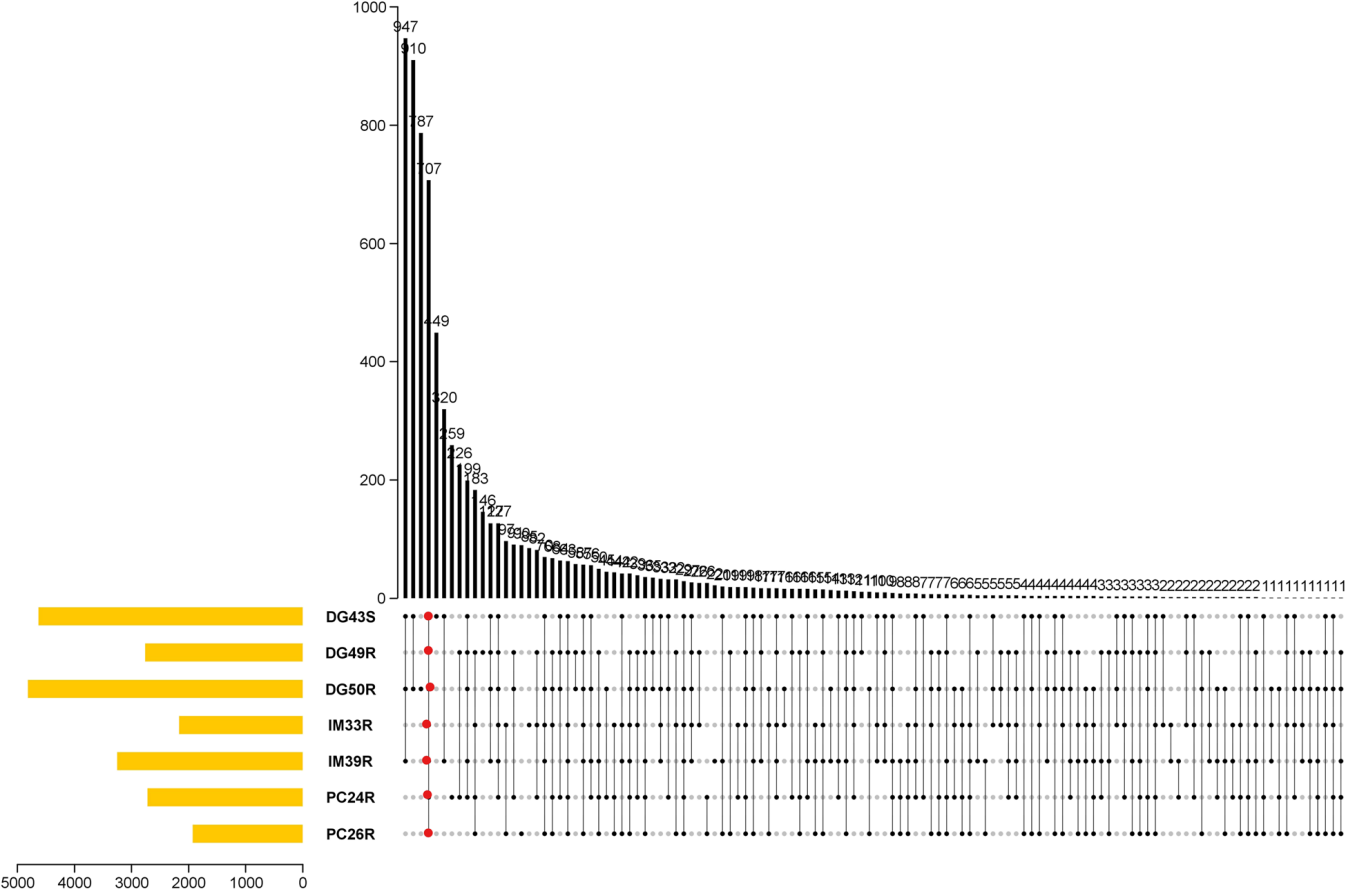
Number of co-regulated transcripts in seven transcriptome libraries prepared from roots harvested from trees with symptoms of citrus blight. Red dots denote a set of 707 transcripts that were co-regulated within all seven libraries. Black dots denote numbers of co-regulated transcripts in the respective libraries. The gold bars indicate the total number of transcripts that were differentially regulated with respect to the healthy control in each library

Each of the RNA-Seq technical replicate libraries was on average 7.25 Mb composed of 71.8 million reads, with 93.6% of reads with Q30 scores greater than 30 and mean quality of 35.55 (Additional file 1: Table S1). Two paired-end reads defined each fragment and four technical replicate libraries were pooled to become the final libraries for each of the eight trees tested (Table 1). The *Citrus sinensis* transcripts were annotated using the sweet orange genome [52] and assigned to different Mapman functional bins by Mercator 3.6 (Additional file 2: Figure S1). Although precautions were taken to limit RNase activity during sampling and extraction of RNA from affected roots, the RNA integrity number (RIN) [44] for the seven libraries varied from 1 to 8. The percentage of RNA fragments that were successfully mapped to the citrus genome also varied from 42.6–95.1% (Table 1). When the seven libraries were integrated, 707 transcripts were co-regulated as compared to the control (Table 2, Fig. 2). The general patterns of expression were highly consistent in all seven libraries.

**Table 2:** Summary of differentially regulated transcripts in roots of trees affected by citrus blight

Library	Number of transcripts		
	Up	Down	Total
IM33R	501	1504	2005
DG49R	686	1934	2620
PC24R	708	1906	2614
PC26R	411	1336	1747
DG43S	1008	3397	4405
DG50R	904	3593	4497
IM39R	891	2193	3084
Co-Regulated	76	631	707

**Fig. 3:**
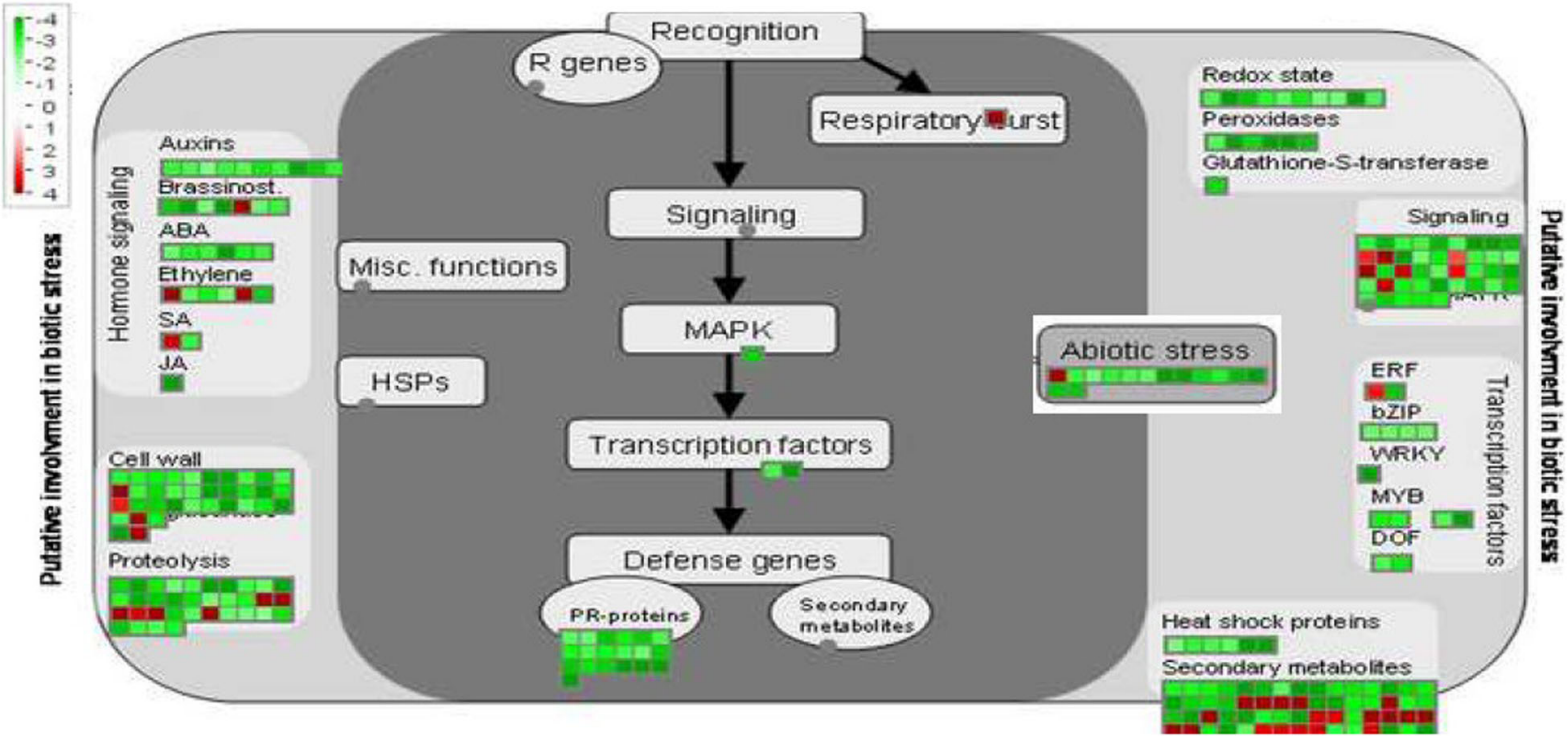
Biotic responses of co-differentially expressed transcripts within seven RNA-Seq libraries from trees roots affected by citrus blight

Within the seven libraries, the co-differentially expressed transcripts (DETs) related to the biological categories of metabolic processes (GO:0008152) and cellular processes (GO:0009987) were most disrupted, followed by response to stimulus (GO:0050896) (Additional file 3: Figure S2A). Within the cellular component category, subcategories associated with cell parts, membranes and organelles were heavily affected (Additional file 3: Figure S2B). The metabolic pathways that were most heavily affected by citrus blight included pathways related to the cell wall, hormone signaling, proteolysis, transcription factors, and secondary metabolism. Pathogenesis related (PR-protein) genes and associated pathways were strongly affected and overwhelmingly repressed (Fig. 3).

### Primary and secondary metabolism

In all seven libraries, co-DETs involved in primary metabolism, including carbohydrate, lipid, amino acid and protein, as well as secondary metabolism were overwhelmingly down-regulated (Additional file 4: Table S2). Both sucrose and starch metabolism were especially downregulated (Additional file 4 Table S2 Bin 2.1.1.1). Transcripts of TPS11 (trehalose phosphatase/synthase 11 (Additional file 4 Table S2 Bin 3.2.3) were up-regulated, whereas GSL12 (glucan synthase-like) and five cellulose synthase genes were sharply down-regulated (Additional file 4 Table S2 Bin 10.2). This expression pattern was opposite of what was found in response to HLB [20]. Genes encoding PRP4 (proline-rich protein), XTR4 (xyloglucan endotransglycosylase-related protein) and pectin acetyl esterase protein, both involved in cell wall modification were also induced (Table 3). Concanavalin A-like lectin protein involved in post translational modification was also induced (Table 3). Genes encoding a ubiquitin E3 RING zinc finger protein, an aspartyl protease and AATP1 (AAA-ATPase 1), associated with protein degradation in response to stress were also up-regulated (Table 3).

**Table 3:** Differentially regulated transcripts in seven libraries of tree roots affected by citrus blight

Gene_symbol	*Citrus sinensis*_ID	Description	Ave. FC (log2)
Carbohydrate metabolism and Cell Wall			
TPS11	cs4g02730	Encodes an enzyme putatively involved in trehalose biosynthesis	3.9
GSL12	orange1.1 t02029	glucan synthase-like 12,similar to callose synthase	−3.0
CSLB04	cs1g03980	cellulose synthase-like B4	−3.0
CESA6/CESA9	cs1g04570	cellulose synthase, related to CESA6/cellulose synthase A9	−2.5
CSLB06	cs2g21100	cellulose synthase-like B6	−4.2
CESA6I	cs3g21530	a cellulose synthase isomer. CESA6	−3.9
CESA3I	cs7g01740	a cellulose synthase isomer. CESA3	−3.0
PRP4	cs8g09090	Encodes one of four proline-rich proteins	4.3
XTR4	cs3g08950	xyloglucan endotransglycosylase-related protein 4	3.0
ConA-like	cs8g12370	Concanavalin A-like lectin protein kinase	4.5
PAE	orange1.1 t03602	Pectinacetylesterase family protein	4.4
AAE12	cs6g15880	acyl activating enzyme 12	3.4
AMP-dependent	cs9g17340	AMP-dependent synthetase and ligase family protein	3.3
E3.RING	cs2g10580	Zinc finger (C3HC4-type RING finger) family protein	4.4
NA	cs9g06040	Eukaryotic aspartyl protease family protein	3.9
AATP1	cs6g20470	AAA-ATPase 1	4.1
Secondary Metabolism			
CAD	cs1g20580	NAD-dependent mannitol dehydrogenase	6.1
COMT	orange1.1 t05018	Caffeic acid 3-O-methyltransferase	−4.1
OMT1	cs5g16860	A caffeic acid/5-hydroxyferulic acid O-methyltransferase	6.7
AAE12	cs6g15880	acyl activating enzyme 12	3.4
UGT74E2	cs2g18290	Encodes a UDP-glucosyltransferase	3.5
CYP79A2	cs7g29740	Encodes cytochrome P450 79A2	4.3
CYP79A2	cs7g29760	Encodes cytochrome P450	5.0
CYP79A1	cs7g29750	Cytochrome P450 79A1	4.7
Transport			
PDR3	cs8g20130	pleiotropic drug resistance 3	2.5
AMT2	cs6g08950	ammonium transporter 2	3.6
AMT1;1	orange1.1 t03479	ammonium transporter 1;1	3.2
PHT3;1	cs7g19830	phosphate transporter 3;1	3.7
PIP1.1	cs6g07970	Aquaporin PIP1.1	−2.9
PIP1	cs7g31410	a member of the plasma membrane intrinsic protein subfamily PIP1	−4.0
PIP3	cs8g02530	a member of the plasma membrane intrinsic protein PIP3	−3.6
PIP2.2	cs8g16640	Probable aquaporin PIP2.2	−5.2
TIP	cs1g15440	Delta tonoplast intrinsic protein	−5.6
TIP	orange1.1 t03005	Encodes a tonoplast intrinsic protein, functions as water channel	−4.0
EXO70	cs6g05440	A member of EXO70 gene family, involved in cell vesicle transport	3.6
MRP-like ABC	cs7g10200	an ATP-dependent MRP-like ABC transporter	3.4
NA	cs1g01440	Major facilitator superfamily protein	2.4
NA	cs3g27810	Auxin efflux carrier family protein	3.8
NA	cs9g06620	Encodes a protein with hexose-specific/H+ symporter activity	4.0
NA	cs1g01440	Major facilitator superfamily protein	2.4
Hormone and Plant Defense Responses			
LRR repeat	cs5g11310	Leucine-rich repeat protein in brassinosteroid signal transduction	4.6
2OG-Fe (II)	cs7g12100	2-oxoglutarate (2OG) and Fe (II)-dependent oxygenase	4.2
ACCO1	cs2g20590	1-aminocyclopropane-1-carboxylate oxidase 1	5.4
UGT74E2	cs2g18290	Encodes a UDP-glucosyltransferase	3.5
ChiA	cs8g01840	Endochitinase A precursor	−7.9
DOX	cs2g28680	Encodes an alpha-dioxygenase	5.1
RbohD	cs8g12000	NADPH/respiratory burst oxidase protein D	4.0
Kunitz	cs5g13890	Kunitz family trypsin and protease inhibitor protein	−10.9
B120	cs2g07100	protein serine/threonine kinase activity	3.6
CDRK1	cs6g17020	CDPK-related kinase 1	−3.3
calcineurin B-like	cs1g18400	Encodes a member of the calcineurin B-like calcium sensor gene	−2.8
MuDR	orange1.1 t00859	MuDR family transposase	3.1
TNT 1–94	cs2g17600	Retrovirus-related Pol polyprotein from transposon TNT 1–94	−3.5
TNT 1–94	cs1g01170	Retrovirus-related Pol polyprotein from transposon TNT 1–94	2.7
TNT 1–94	cs3g09730	Retrovirus-related Pol polyprotein from transposon TNT 1–94	−2.8
WAK	cs1g13910	Encodes a receptor-like kinase	3.3
ATPase E1-E2	cs5g30640	ATPase E1-E2 type/haloacid dehalogenase-like hydrolase protein	3.7
PIK	cs6g19820	Phosphoinositide kinase	−3.6
PXY	cs9g14980	Phloem intercalated with xylem	−3.8
LRR protein	cs9g14860	Leucine-rich repeat receptor-like protein kinase family protein	4.2
NA	orange1.1 t00778	S-adenosyl-L-methionine-dependent methyltransferases	4.2
NA	cs8g02010	Transmembrane amino acid transporter family protein	3.7
Not Assigned			
PLAC8	cs2g17160	Protein of unknown function Cys-rich	4.2
NA	orange1.1 t02977	HXXXD-type acyl-transferase family protein	3.7
NA	orange1.1 t02980	HXXXD-type acyl-transferase family protei	3.9
NA	cs1g19935	not assigned.unknown	3.2
NA	cs3g04670	not assigned.unknown	4.2
NA	cs3g16240	not assigned.unknown	4.3
NA	cs3g21660	not assigned.unknown	5.2
NA	cs4g18120	not assigned.unknown	3.4
NA	cs6g11300	not assigned.unknown	4.3
NA	cs6g15820	not assigned.unknown	4.0
NA	orange1.1 t03318	not assigned.unknown	4.6
NA	orange1.1 t03320	not assigned.unknown	3.9

Ave. FC, average fold change of seven libraries

NA, no gene symbol

In contrast to the cellulose synthase genes that were downregulated, two genes involved in lignin biosynthesis and a suite of transcripts encoding aromatic monooxygenase CYP79A2 for sulfur containing aromatic glucosinolates were very sharply upregulated (Table 3; Additional file 4 Table S2 Bin 16.5.1.1.2).

### Energy and transport

Genes for mitochondrial electron transport and ATP synthesis via the NADH-DH complex were consistently down regulated (Additional file 4: Table S2 Bin 9.1) consistent with a reduced capacity for generation of energy. This may have contributed to a reduced capacity of the transportation system in the root phloem. Substances transported in the phloem include sugar, peptides, amino acids, potassium and metal ions. Notably, both major intrinsic proteins PIP (plasma membrane intrinsic proteins) and TIP (tonoplast intrinsic protein) with water channel activity were down-regulated (Table 3). The repression of PIP1.1, PIP2.2 and TIP in the phloem is consistent with reduced water movement. Overexpression of ammonium transporters AMT2, AMT1;1 and phosphate transporter 3;1 was observed (Table 3; Additional file 4: Table S2 Bin 34.5). The expression of two ABC transporters and a multidrug efflux protein involved in cross membrane movement of substances were induced. The major facilitator superfamily of peptide and oligopeptide transporters were down regulated, but many more transcripts of genes for the transport of peptides and oligopeptides were up regulated (Additional file 4 Table S2 Bin 34.13).

### Hormones and plant defense responses

Metabolic pathways associated with abscisic acid, auxin, brassinosteroids, cytokinin, ethylene, gibberellin jasmonate and salicylate were all overwhelmingly repressed with induction of only ACCO1 (1-aminocyclopropane-1-carboxylate oxidase 1), used to produce ethylene (Additional file 4: Table S2 Bins 17.1–17.6). Genes associated with a wide range of transcription factors (Additional file 4 Table S2 Bin 27), and response to biotic and abiotic stresses (Additional file 4: Table S2 Bin 20) and signaling receptors were also predominantly repressed in all seven libraries. Transcripts encoding a MuDR transposase, participating in the regulation of transposition were consistently induced, but transcripts encoding different retrovirus-related polyproteins were inconsistently regulated either up or down (Table 3). Calcium-dependent protein kinase 1 (CDPK1) and calcineurin B-like protein functioning in calcium signaling and phosphoinositide kinase in phospholipid signaling were repressed (Table 3). A few receptors, representing legume-lectin, S-locus glycoprotein, wall associated kinase, ATPase E1-E2 and leucine-rich repeat, play roles in signaling were induced (Table 3). Notably, genes encoding Kunitz-type protease inhibitor and endochitinase A precursor were extremely down-regulated. There was also a set of up-regulated genes of unknown function that were not assigned into bins (Table 3).

### Correlation between RNA-Seq data and RT-qPCR

**Fig. 4:**
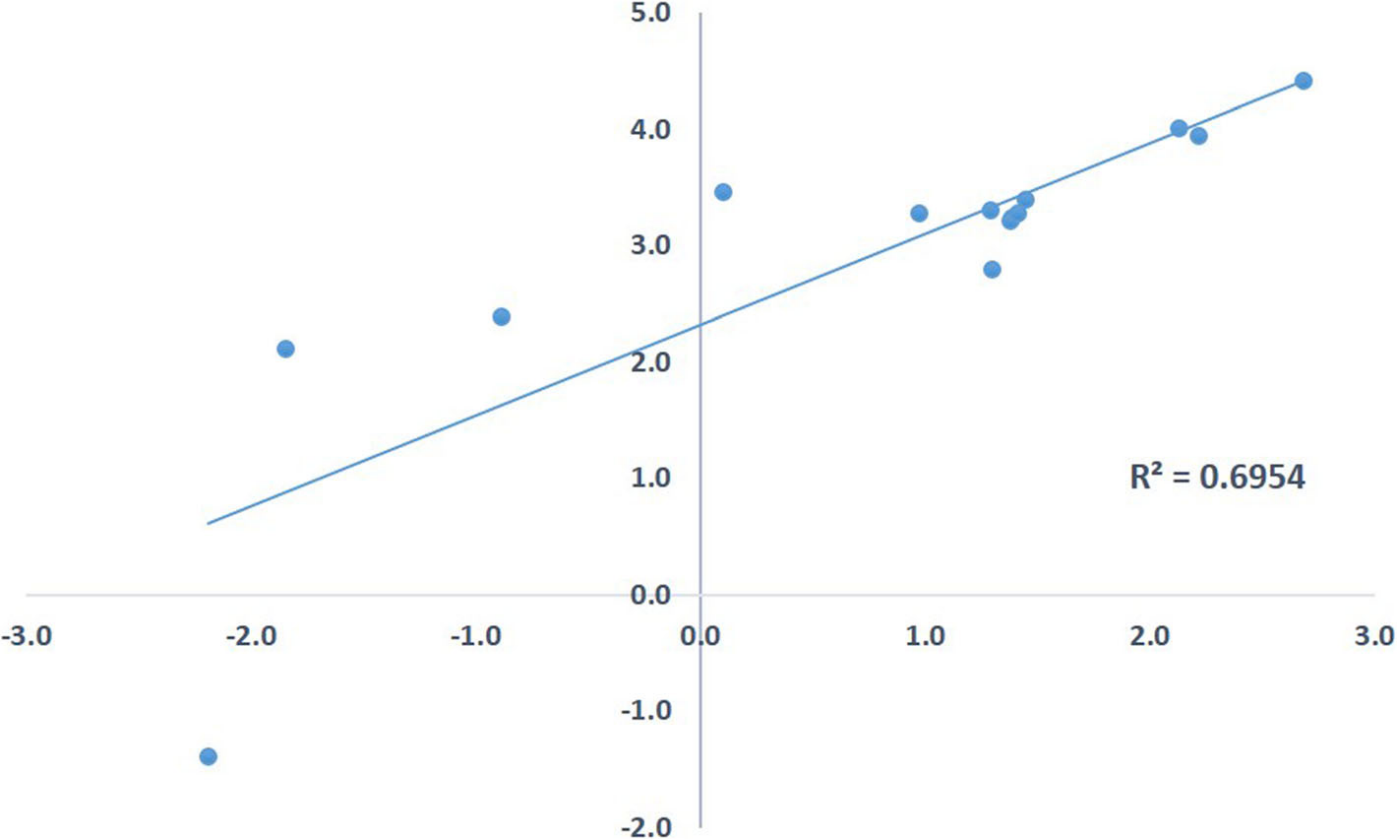
Spearman correlation values between RNA-seq and qPCR for seven root samples from trees affected by citrus blight and transcripts of polyprotein and disease related transcripts

The level of expression of genes encoding transcripts annotated as disease resistance/polyprotein/citrus endogenous pararetrovirus was estimated by both RNA-Seq and RT-PCR (Additional file 5 Table S3). The correlation of the two techniques was evaluated by the Spearman’s rho value, which 0.70 for all seven libraries (Fig. 4).

### Presence of genomic sequences from other organisms in the libraries

We examined the sequence libraries for evidence of other organisms that may be associated with citrus blight disease (Table 4). As expected, the vast majority of the reads were from citrus, but all of the libraries also had reads from plant viruses, notably citrus tristeza virus. The libraries from the roots of trees with citrus blight also had significant numbers of reads mapped to pararetrovirus, CBaPRV, similar to pararetroviruses that we have described earlier from citrus blight infected trees [42]. We also observed large number of reads that mapped to *Pseudomonas spp.* Bacteria in the genus Pseudomonas have been observed previously in association with citrus blight [21, 22]. Sequences of *Xylella fastidiosa* were not observed.

**Table 4:** Distribution of fragments of mapped reads to the citrus and other genomes

Library	Total	All Plant Virus	CBaPRV	Citrus genome	Mitochondria	Pseudomonas	Unmapped
HEALTHY	148,237,895	16,209	13,408	94,130,532	10,326,229	3887	43,447,630
IM33R	136,912,322	1,038,343	61,593	71,680,596	16,322,008	1,092,160	46,717,622
DG49R	140,225,261	1,902,459	18,633	82,661,128	19,205,633	46,467	36,390,941
PC24R	137,259,789	1,303,002	14,017	90,557,581	12,524,404	104,470	32,756,315
PC26R	141,882,978	490,461	65,743	80,535,945	10,820,785	891,220	49,078,824
DG43S^a^	137,256,704	264,600	111,012	56,240,983	20,178,634	5343	26,000,498
DG50R	138,399,721	49,072	147,522	93,983,664	13,060,335	587,965	30,571,163
IM39R	137,256,704	574,979	103,402	75,571,692	17,757,232	1,334,509	41,914,890

^a^ One of four technical replicates in library DG43S had a corrupt read that caused that replicate to not map properly. Therefore, only three of four technical replicates, or 75% of the total reads were mapped for DG43S

**Fig. 5:**
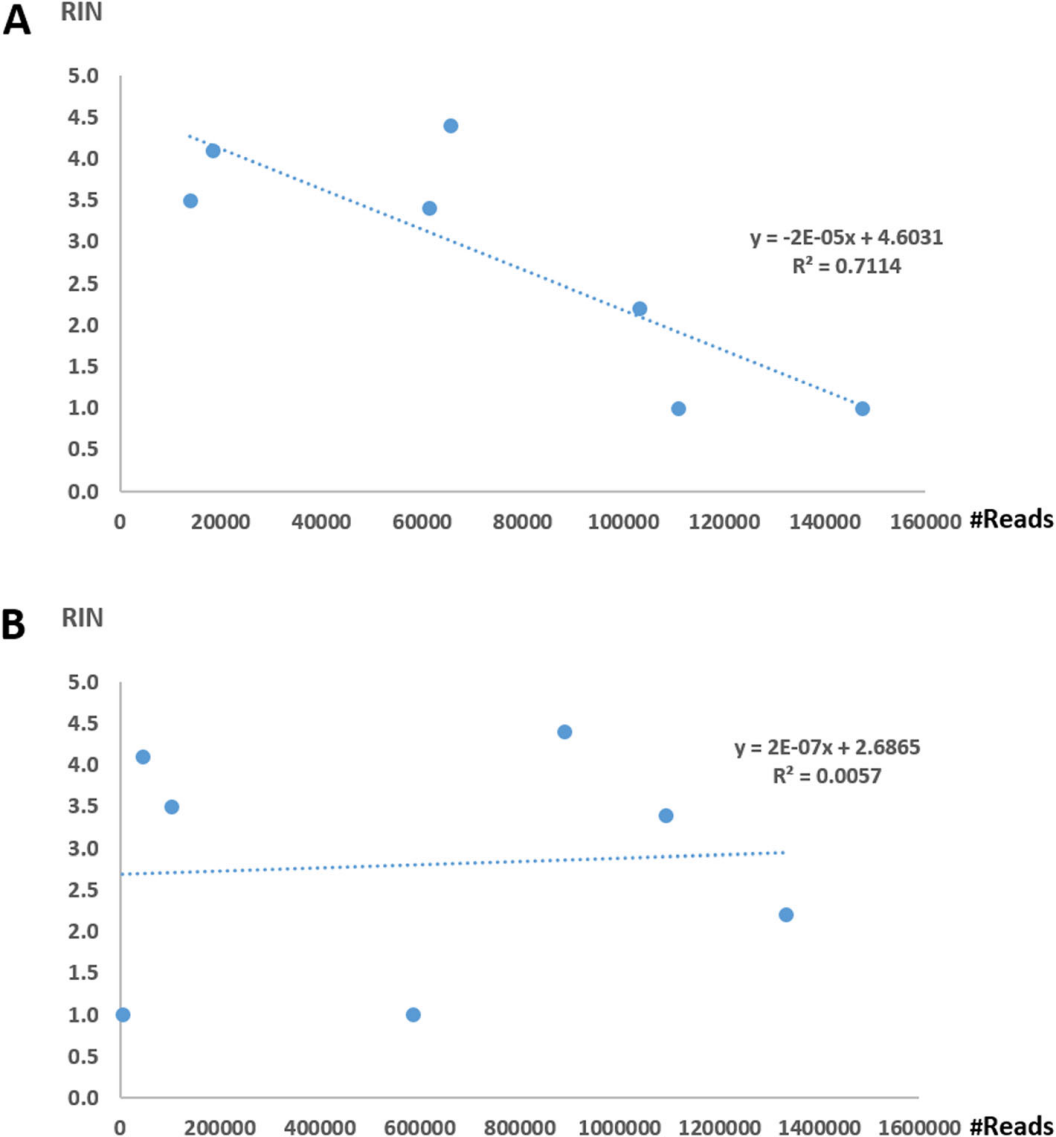
Correlation between reads mapped to CBaPRV or Pseudomonas and RIN. **a.** The number of transcript reads mapped to CBaPRV in libraries prepared from RNA extracts prepared from seven root samples from trees affected by citrus blight vs the RNA Integrity Number. **b.** The number of transcript reads mapped to Pseudomonas vs the RNA Integrity Number in the same RNA libraries

The quality of the RNA used to make the transcript libraries was variable when the libraries were prepared from blight affected trees, but not from the healthy control tree (Table 1). The quality of the RNA library was inversely correlated with the number of reads that mapped to CBaPRV in each of the libraries (R^2^ = 0.7114; Fig. 5a). There was no such correlation when the RIN was plotted against the number of reads that were mapped to Pseudomonas in the same libraries (R^2^ = 0.0057; Fig. 5b).

## Discussion

Many more genes were down- than up-regulated in response to citrus blight, accounting for 89% of the total number of genes whose expression was altered in the seven libraries, in general agreement with an earlier study [55]. In contrast with the earlier study, trees sampled in this study had fully developed citrus blight with symptoms of die back, foliar zinc deficiency and severely impaired water uptake.

Xylem plugging leading to impaired transport of water is a physical change in the plants with blight. Over production of proline-rich proteins could help citrus plant cells to maintain water potential, thus enhancing tolerance to water stress as observed in transgenic tobacco [25]. Tobacco transformed with trehalose phosphate synthase (TPS1) and trehalose-6-phosphate synthase, exhibited drought tolerance [5] and improved photosynthetic efficiency, respectively under drought [26]. Presumably, the induction of trehalose assists the citrus plant adapt to dehydration caused by blight.

As a consequence of water loss, modifications of protein residues such as deamination or oxidation result in the disruption of native protein conformation or protein activity. The tree responds to this by activating the ubiquitination process and proteases to remove the damaged proteins and recycle the amino acids. Concanavalin A-like lectin protein (Con A-like) is involved in phosphorylation and alterations in protein phosphorylation suggest reversible protein phosphorylation is being used by the orange trees as a regulator in response to water deficit [15]. Thus, up-regulation of Con A-like could favor repair of damage in the plants. OMT1, CYP79A1, CYP79A2 and CYP79A9 proteins function in the biosynthesis of lignin and glucosinolates and their overexpression is likely to be associated with lignin and amorphous plugging in the xylem vessels [34, 51].

Aquaporins, PIP (plasma membrane intrinsic proteins) and TIP (tonoplast intrinsic protein), are water channel proteins that facilitate water transport along transmembrane water potential gradients [39] and their down-regulation is consistent with reduced water transport in the CB plants. Roots from trees suffering from citrus blight were previously shown to experience nitrogen starvation, while nitrogen accumulated in the trunk wood, bark, and leaf tissue. This could be consistent with the up-regulation of transporters for ammonia. The repression of MDR1 possibly reflected the impairment of IAA gradient-driven transport in the roots, consistent with the decrease of IAA oxidase activities in declining trees. The repression of transcripts encoding zinc transporters is consistent with the accumulation of zinc in the roots as observed in the trunk phloem [3, 4] and would contribute to the foliar symptoms of zinc deficiency since the ability to mobilize zinc is diminished. CB-affected trees also exhibit visible symptoms of wilting and general lack of flush [41, 54]. CDPKs and calcineurin B-like proteins effect calcium signaling and gene expression in response to drought [31, 50]. Their repression, along with phosphoinositide kinase indicated that calcium and phospholipid signaling pathways were blocked and thus amplification of the water stress signal after its perception was likely impaired. This is consistent with the negative regulation of nucleic acid, carbohydrate, lipid, protein cell organization and development associated pathways as well as a lack of water and demand for new growth, consistent with the earlier report [55]. Our data indicate that phloem and xylem function were heavily disturbed with accompanying unbalanced ion homeostasis under blight stress.

It is especially notable that cellulose synthesis in the cell wall was sharply down regulated in roots of trees affected by CB. At the same time, biosynthetic pathways that lead to lignin production were sharply upregulated. In a healthy tree, cellulose fibers and lignin molecules are assembled into wood. It appears that in trees affected by CB the balance between cellulose and lignin production is perturbed leading to an accumulation of soluble lignin in xylem vessels [51], which could lead to the plugging of xylem vessels observed. Although water cannot be forced into the trunks by syringe injection of trees affected by CB, the trees do not die of wilt, but suffer dieback, lack of flush and fail to thrive. The uptake of water in blighted trees has been experimentally determined by gravity injection to be in the range of 5–43 ml/day compared to 151–503 ml/day among the healthy control trees [12]. Thus, the transpiration stream is severely impaired rather than completely stopped.

Although the trees are very clearly under water stress, the expression of genes related to abscisic acid were not uniformly regulated either up or down, in contrast with results of trees at earlier stages of CB [55]. Expression of ACCO1 synthase was increased consistent with a generalized stress response, also in contrast with what was seen at earlier stages of the CB. The increased activity of the ubiquitin pathway for protein degradation suggests that the roots of trees with citrus blight process a great deal of denatured and damaged proteins which would otherwise support necessary physiological processes. Accelerated degradation of proteins would explain the general up regulation of transporters of oligopeptides and ammonium as the tree attempts to maintain protein homeostasis. Taken together, the data explain why trees with citrus blight are not productive but may linger for years in a ‘zombie-like’ state.

Transcript libraries can be mined to discover signature sequences to identify unknown organisms such as viruses that are associated with plant diseases of unknown etiology [30]. Sequences from non-citrus genomes were found in the libraries from trees affected by citrus blight. CTV is now ubiquitous in Florida and so is expected in the libraries but is not associated with citrus blight because citrus blight was present in Florida for nearly a century before CTV was introduced to the United States [7, 41]. There are large numbers of retrotransposon and pararetrovirus-related sequences in the sweet orange genome [52] and some are located at a locus that confers resistance to CTV [53] and may be expressed in response to stressful conditions such as occur in citrus blight. The sequence read libraries were mapped to the reference genome of the CBaPRV before they were mapped to the citrus genome to account for the presence of these pararetrovirus sequences in the citrus genome. The number of reads of the CBaPRV were elevated in libraries prepared from roots of the blight affected trees (Table 4). Pseudomonads have been reported previously in association with citrus blight [21, 22] and are likely to be present as opportunists. *Xylella fastidosa* was proposed as an etiologic agent for citrus blight [27, 28] but was not present in our samples.

We have previously observed pararetrovirus sequences in small RNA libraries such as used for this study [43]. It is interesting that the number of CBaPRV reads present in the libraries was strongly and inversely correlated with the quality of the RNA in the libraries. Such a correlation was not observed for reads that mapped to Pseudomonas in similar numbers. Pseudomonads have been found in association with roots of trees with citrus blight [21, 22] but their association with the roots is not likely to be related to citrus blight and they were not correlated with poor RNA quality as was CBaPRV.

## Conclusion

Water deficit and xylem plugging are two core physical changes under blight stress and they are primarily responsible for the overwhelming repression of genes and disrupted pathways. Down regulation of cellulose production and up regulation of lignin production creates an imbalance for production of wood. The plants altered regulation of both primary and secondary metabolism, transport and hormone associated pathways but failed to reverse the citrus blight syndrome, which is the result of the co-ordination of physiological and biochemical alterations at the cellular and molecular level.

## Materials and methods

### Plant materials, RNA extraction and sequencing

We used a direct water uptake assay in citrus trunks [33] to identify symptomatic trees from commercial groves located in four different growing regions (Collier, DeSoto, Lake and Polk counties) in Florida. Scaffold root samples (~ 1.5 cm diameter) from field trees that failed to take up water and healthy root samples from a 4-year old greenhouse-grown tree ‘Navel orange’ were collected. A greenhouse-grown tree was used as the healthy control because citrus blight does not occur in greenhouse grown trees and the incidence of blight in the groves sampled was such that an apparently healthy tree used as a control may in fact have been in early stage of citrus blight. Roots were placed on ice immediately after harvest and taken to the laboratory. Roots were washed and phloem-enriched tissues (bark) were scraped with a razor blade from roots and stored in RNA-Later (Life Technologies) at −20C. Aliquots of the phloem-enriched samples were ground in liquid nitrogen and RNA was extracted using the Trizol Reagent (Life Technologies). RNA quantity and quality was assessed with a Qubit 2 Fluorimeter (Life Technologies) and a Bioanalyzer 2100 [24]. The eight RNA extracts, seven blight samples and one healthy, were sent out for 2 X 100 bp HiSeq 2500 sequencing (Genewiz, Inc., South Plainfield, NJ).

In previous analyses of root samples from trees affected by citrus blight, 21 nt and 24 nt peaks following RNA-Seq were analyzed [42, 43]. Several transcripts annotated as polyprotein/citrus endogenous pararetrovirus and disease resistance were found to have altered expression in response to citrus blight. Consistent expression changes of the tested genes were observed in the seven libraries from CB affected trees.

### RT-qPCR assay of transcripts of polyprotein and disease related genes

A total of 3 μg of RNA was reverse transcribed for first-strand cDNA syntheses with GoTaq® 2-step RT-qPCR system (Promega, Madison, WI) following the manufacturer’s instructions. cDNA was diluted three-fold with 1 X TE and stored at -20 °C for use. Gene-specific primers (Additional file 5: Table S3) were designed with an online tool (Integrated DNA Technologies, Coralville, IA) with melting temperatures of 60 °C ± 5 °C. qPCR reactions were performed with GoTaq® qPCR Master Mix (Promega) in a Bio-Rad CFX96 system. Twenty microliters of reaction mixture were added to each well with three replicated plant samples for each primer pair for each sample. The amplification program was set at 95 °C for 3 min, and 40 cycles of 95 °C for 10 s, 60 °C for 30 s and 72 °C for 30 s. Melting curves were analyzed to ensure that a single product was amplified. The GAPC2 gene has been verified to be a stable reference gene [36] and was used as the internal control in our study. The 2^-△△Ct^ method was applied for relative quantification [35].

### Statistical analysis

One RNA sample from each of 8 trees was run in each of 4 lanes as technical replicates in an Illumina 2500 system to produce 32 libraries of paired-end reads (2 × 100 nucleotides). Raw sequence reads were trimmed to remove possible adapter sequences and nucleotides with poor quality (error rate < 0.05). After trimming, sequence reads shorter than 50 nucleotides were discarded (Genewiz). The trimmed reads were then mapped to the coding sequences of the reference genome *Citrus sinensis* 2.0 [52] with Bowtie2 [32] (Table 1). The 4 technical replicates were pooled and the expression level of transcripts (log_2_ fold-change) were calculated with GFOLD [18], which evaluates the statistical significance of fold-change values against the Poisson distribution rather than the normal distribution. Differentially expressed transcripts (DETs) were normalized as reads per kilobase per million reads (RPKM) with cut-off values, reads ≥200 and ∣ log_2_FC ∣ ≥ 2, *P* < 0.01. The functions of DETs were enriched by mapping to *C. sinensis* 2.0 with Mapman [45]. The mapping file was generated by Mercator using PlaBi as the reference database (http://www.plabipd.de/portal/web/guest/home1). Upset diagrams of co-regulated DETs of the seven libraries were created with TBtools [13]. DETs were also classified by alignment with *Arabidopsis thaliana* (e-value ≤ e-4) using Panther (http://pantherdb.org) [40].

### Mapping of transcript reads to the sweet orange and other genomes

The raw sequence reads were pruned and cleaned using BBDuk version 37.66 with adapters.fa from the BBTools software suite [10]. The technical replicates were then pooled. The reads were mapped using Bowtie2 version 2.3.4 with default parameters [32] and 20 threads to reference genomes as follows: The reads were first mapped to All Plant Virus using the DPV web database [1], then to the CBaPCRV (NCBI XXXXXXX), then to the citrus genome (CGF_00317415) then to a large number of strains of *Pseudomonas syringae* and *P. fluorescens* such as (NC_004578.1, NC_004633.1, NC_004632.1, NC_007005.1, NC_007005.1) and finally to citrus mitochondria (NC_039463.1). After each mapping step, the remaining unmapped reads were used as input for Bowtie2 (Table 4).

## Supplementary information

**Additional file 1 : Table S1.** Summary of reads and quality scores for a typical lane (technical replicate) of Illumina paired end reads used in this analysis.

**Additional file 2 : Figure S1.** Differentially expressed *Citrus sinensis* transcripts in tree roots in response to citrus blight assigned to bins of Mapman by Mercator.

**Additional file 3 : Figure S2**. Distribution of co-differentially expressed transcripts within the (**A**) Biological Process and (**B**) Cellular Component gene ontology categories. Transcripts were from all seven RNA-Seq libraries from tree roots with symptoms of citrus blight.

**Additional file 4 : Table S2**. Differentially expressed *Citrus sinensis* transcripts in tree roots in response to citrus blight assigned to bins of Mapman by Mercator. Some of the 707 differentially regulated transcripts were placed in more than one bin.

**Additional file 5 : Table S3**. Transcript targets and primer information for RT-qPCR assays.

## Data Availability

The datasets supporting the conclusions of this article are available in the SRA databasae under Submission ID XXXXXXXXXXX and Bioproject ID YYYYYYYYYYYYY; https://www.ncbi.nlm.nih. CBaPRV sequence is deposited at NCBI Genbank as accession number XXXXXX.

## Abbreviations

CB: Citrus blight
DET: Differentially expressed transcripts
HLB: Huanglongbing
RIN: RNA integrity number
