# Supplementary material for: Transcriptomic analyses reveal physiological changes in sweet orange roots affected by citrus blight
Source: BMC Genomics. 2019 Dec 11;20:969. doi: 10.1186/s12864-019-6339-0 (PMC6907255; doi:10.1186/s12864-019-6339-0)
Supplement: Supplementary file 1 — Additional file 1 : Table S1. Summary of reads and quality scores for a typical lane (technical replicate) of Illumina paired end reads used in this analysis. [file 12864_2019_6339_MOESM1_ESM.docx]

**Table S1.** Summary of reads and quality scores for a typical lane (technical replicate) of Illumina paired end reads used in this analysis.

| Sample | Yield (Mbases) | # Reads | %>= Q30 | Mean Quality |
| --- | --- | --- | --- | --- |
|  |  |  |  |  |
| All | 57,984 | 574,091,762 | 93.58 | 35.55 |
|  |  |  |  |  |
| HC | 7,711 | 76,341,864 | 93.47 | 35.53 |
|  |  |  |  |  |
| IM33R | 7,089 | 70,185,338 | 94.07 | 35.64 |
|  |  |  |  |  |
| DG49R | 7,285 | 72,131,296 | 93.67 | 35.56 |
|  |  |  |  |  |
| PC24R | 7,121 | 70,504,908 | 93.62 | 35.56 |
|  |  |  |  |  |
| PC26R | 7,385 | 73,114,084 | 93.25 | 35.48 |
|  |  |  |  |  |
| DG43S | 7,110 | 70,392,454 | 93.48 | 35.52 |
|  |  |  |  |  |
| DG50R | 7,163 | 70,921,850 | 93.57 | 35.55 |
|  |  |  |  |  |
| IM39R | 7,120 | 70,499,968 | 93.53 | 35.53 |
