# Supplementary figures and images for: Transcriptomic analyses reveal physiological changes in sweet orange roots affected by citrus blight
Source: BMC Genomics. 2019 Dec 11;20:969. doi: 10.1186/s12864-019-6339-0 (PMC6907255; doi:10.1186/s12864-019-6339-0)

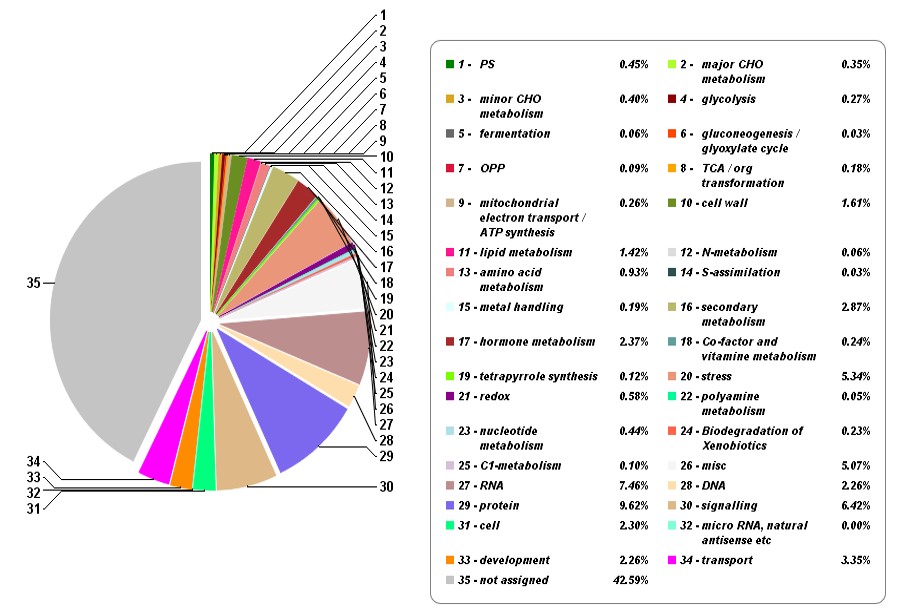

Supplement: Supplementary file 2 — Additional file 2 : Figure S1. Differentially expressed Citrus sinensis transcripts in tree roots in response to citrus blight assigned to bins of Mapman by Mercator. [file 12864_2019_6339_MOESM2_ESM.jpg]

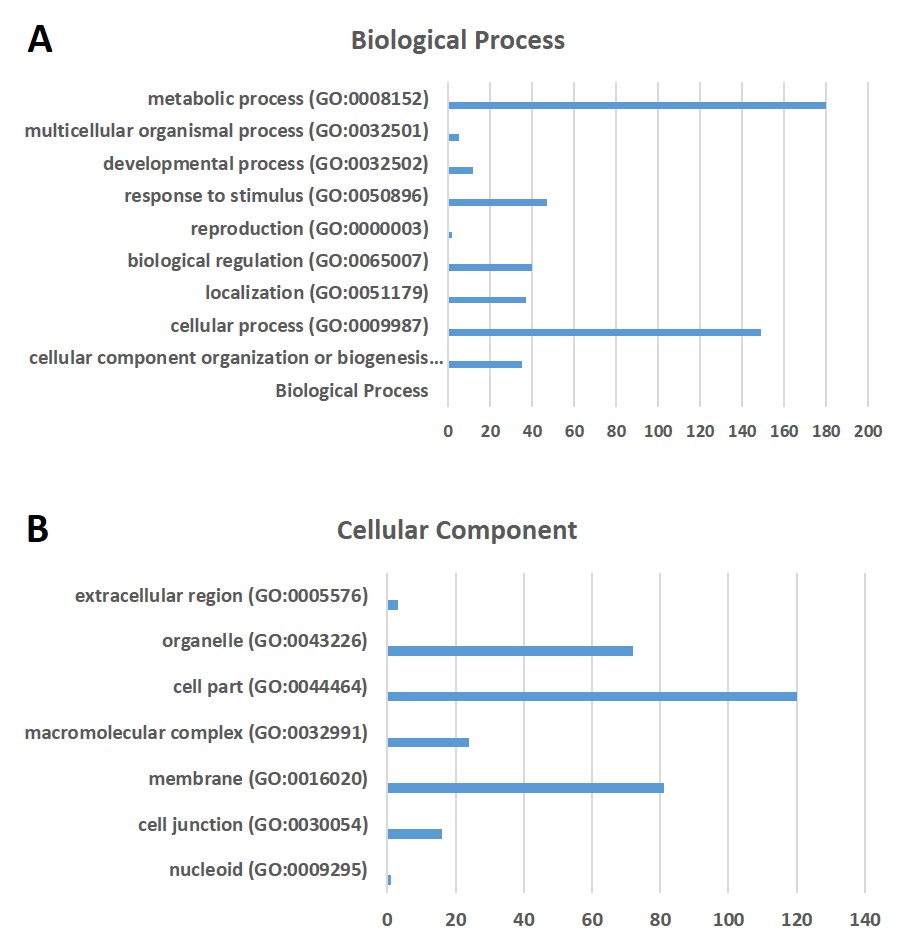

Supplement: Supplementary file 3 — Additional file 3 : Figure S2. Distribution of co-differentially expressed transcripts within the (A) Biological Process and (B) Cellular Component gene ontology categories. Transcripts were from all seven RNA-Seq libraries from tree roots with symptoms of citrus blight. [file 12864_2019_6339_MOESM3_ESM.jpg]
